# Expression of SDF-1*α* and nuclear CXCR4 predicts lymph node metastasis in colorectal cancer

**DOI:** 10.1038/sj.bjc.6604363

**Published:** 2008-04-29

**Authors:** N Yoshitake, H Fukui, H Yamagishi, A Sekikawa, S Fujii, S Tomita, K Ichikawa, J Imura, H Hiraishi, T Fujimori

**Affiliations:** 1 Department of Surgical and Molecular Pathology, Dokkyo University School of Medicine, 880, Kitakobayashi, Mibu, Shimotsuga, Tochigi, 321-0293, Japan; 2 Department of Gastroenterology, Dokkyo University School of Medicine, 880, Kitakobayashi, Mibu, Shimotsuga, Tochigi, 321-0293, Japan

**Keywords:** chemokines, SDF-1*α*, CXCR4, colorectal cancer, lymph node metastasis

## Abstract

Although stromal cell-derived factor (SDF)-1*α* and its receptor CXCR4 are experimentally suggested to be involved in tumorigenicity, the clinicopathological significance of their expression in human disease is not fully understood. We examined SDF-1*α* and CXCR4 expression in colorectal cancers (CRCs) and their related lymph nodes (LNs), and investigated its relationship to clinicopathological features. Specimens of 60 primary CRCs and 27 related LNs were examined immunohistochemically for not only positivity but also immunostaining patterns for SDF-1*α* and CXCR4. The relationships between clinicopathological features and SDF-1*α* or CXCR4 expression were then analysed. Stromal cell-derived factor-1*α* and CXCR4 expression were significantly associated with LN metastasis, tumour stage, and survival of CRC patients. Twenty-nine of 47 CXCR4-positive CRCs (61.7%) showed clear CXCR4 immunoreactivity in the nucleus and a weak signal in the cytoplasm (nuclear type), whereas others showed no nuclear immunoreactivity but a diffuse signal in the cytoplasm and at the plasma membrane (cytomembrane type). Colorectal cancer patients with nuclear CXCR4 expression showed significantly more frequent LN metastasis than did those with cytomembrane expression. Colorectal cancer patients with nuclear CXCR4 expression in the primary lesion frequently had cytomembrane CXCR4-positive tumours in their LNs. In conclusion, expression of SDF-1*α* and nuclear CXCR4 predicts LN metastasis in CRCs.

Chemokines belong to the small molecular chemoattractive cytokine family and are classified into four groups (CXC, CXC_3_, CC, and C) according to the positions of the four conserved cysteine residues (Baggiolini *et al*, 1997; Rollins, 1997). Their actions are mediated by G-protein-coupled receptors, which are characterised by a seven-transmembrane-spanning domain. Stromal cell-derived factor (SDF)-1*α*, which was originally cloned from murine bone marrow (Tashiro *et al*, 1993), is a member (CXC chemokine ligand 12) of the CXC subfamily and exerts an effect through its specific receptor CXCR4 (Tachibana *et al*, 1998; Zou *et al*, 1998). The SDF-1*α*–CXCR4 axis was initially found to be stimulated by the homing of lymphocytes to inflammatory tissues and has recently been found to be involved in many areas of immunology and human development, including organogenesis, vascularisation, haematopoiesis, and embryogenesis (Murdoch, 2000). Moreover, recent studies have reported that SDF-1*α* and CXCR4 may play important roles in cell survival, proliferation, chemotaxis, migration, and adhesion (Vlahakis *et al*, 2002; Kayali *et al*, 2003; Soriano *et al*, 2003; Fernandis *et al*, 2004; Hartmann *et al*, 2005), suggesting that the SDF-1*α*–CXCR4 axis is involved in tumorigenicity. However, although these biological functions of SDF-1*α* and CXCR4 were examined mainly in *in vitro* studies, the pathophysiological significance of SDF-1*α* and CXCR4 in human disease still remains unclear. Therefore, we examined the expression of SDF-1*α* and CXCR4 in colorectal cancers (CRCs) and their related lymph nodes (LNs), and investigated the relationship between this expression and clinicopathological features. In addition, because our immunohistochemical analysis of CRC cells revealed nuclear expression of CXCR4, which is normally expressed in the cytomembrane, we also investigated the presence of CXCR4 protein in the nucleus and its pathophysiological significance.

## MATERIALS AND METHODS

### Patients and tissue samples

A total of 60 patients with CRC who underwent surgery or endoscopic resection at Dokkyo University School of Medicine between 1990 and 2003 were enrolled. Patients with familial adenomatous polyposis, hereditary nonpolyposis colorectal cancer, inflammatory bowel disease, or other malignancies were excluded, as were patients who had received preoperative treatment such as chemotherapy or radiation therapy. The study was performed with the approval of the Dokkyo University Surgical Pathology Committee, and informed consent was obtained from all patients.

The resected specimens were fixed in 10% formalin and embedded in paraffin. Multiple haematoxylin-and-eosin-stained sections of CRC and its related LNs were examined. The following factors were determined for all patients and lesions: age, gender, tumour location, tumour size, tumour differentiation, tumour invasion, LN metastases, and tumour stage. Tumour differentiation and stage were determined according to the WHO and UICC criteria, respectively. All these clinicopathological features are summarised in Table 1.

### Immunostaining

Immunohistochemical staining for SDF-1*α* and CXCR4 was performed as described previously (Fukui *et al*, 2004). In brief, the sections (4 *μ*m thick) were deparaffinised, rehydrated, placed in 0.01 mol l^−1^ citrate buffer (pH 6.0), and treated by microwave heating for 10 min. The sections were then preincubated with 0.3% H_2_O_2_ in methanol for 20 min at room temperature to quench endogenous peroxidase activity. Subsequently, the sections were immunostained with an UltraTech Kit (Immunotech, Marseille, France) in accordance with the manufacturer's instructions. The sections were pretreated with 1% bovine serum albumin in phosphate-buffered saline (PBS) and then incubated with anti-SDF-1*α* antibody (R&D Systems Inc., Minneapolis, MN, USA; dilution 1 : 50) and anti-CXCR4 antibody (BD Biosciences Pharmingen, San Diego, CA, USA; dilution 1 : 20) for 1 h at room temperature. Thereafter, the sections were incubated with biotinylated secondary antibody for 15 min, washed with PBS, and treated with peroxidase-conjugated streptavidin for 20 min. Finally, the sections were incubated in 3,3′-diaminobenzidine tetrahydrochloride with 0.05% H_2_O_2_ for 3 min and then counterstained with Carazzi's haematoxylin. Sections of oesophageal cancer that had been confirmed to overexpress these proteins were used as positive controls, and antibodies were not applied to negative controls.

### Evaluation of SDF-1*α* and CXCR4 expression

To examine the pathophysiological role of SDF-1*α* and CXCR4 in metastasis, we assessed the immunoreactivity of SDF-1*α* and CXCR4 in the invasive front of CRCs and in their related LN metastases (magnification × 200), because the invasive front is a source of metastasised tumour cells and LNs are the first regions that metastasised tumour cells colonise.

In the present study, we defined the normal endothelial cells as an internal control for SDF-1*α* immunoreactivity. SDF-1*α* immunoreactivity was detected in the cytoplasm of CRC cells. The CRC cells were considered to have strong SDF-1*α* expression if their signal was stronger than or equal to that of endothelial cells in the adjacent normal colonic tissues; otherwise, the CRC cells were considered to have weak SDF-1*α* expression. The CRC samples were classified into a strong group when CRC cells with strong SDF-1*α* expression were dominant at the invasive front of the tumour. Otherwise, we classified them into a weak group.

CXCR4 immunoreactivity was detected in the cytoplasm and in the nucleus of CRC cells. Some CRCs showed clear CXCR4 immunoreactivity in the nucleus and a weak signal in the cytoplasm (nuclear type), whereas others showed no nuclear immunoreactivity but a diffuse signal in the cytoplasm and at the plasma membrane (cytomembrane type). Every lesion was classified as either nuclear or cytomembrane type in accordance with its dominant immunostaining pattern in its invasive front. On the other hand, the CRC samples showing no CXCR4 immunoreactivity were defined as negative.

### Nuclear protein extraction and western blot analysis

A human colorectal cancer cell line, HT29, was maintained in RPMI 1640 medium (Invitrogen, Grand Island, NY, USA) with 10% fetal bovine serum (Sigma Chemical Co., St Louis, MO, USA). Proteins were extracted from these cells and separated into the nuclear fraction and membrane-cytoplasmic fractions, as described previously (Hoshino *et al*, 2007). In brief, the cells were mixed with lysis buffer containing 10 mM Tris-HCl (pH 7.9), 10 mM KCl, 1.5 mM MgCl_2_, 1 mM DTT, 1% Nonidet P-40, and 1 × proteinase inhibitor (Complete Mini; Roche, Mannheim, Germany). Proteins from the membrane and cytoplasm were extracted from the supernatants, and the precipitate was additionally treated with nuclear lysis buffer containing 20 mM Tris-HCl (pH 7.9), 400 mM NaCl, 1.5 mM MgCl_2_, 0.2 mM EDTA, 1 mM DTT, 5% glycerol, and 1 × proteinase inhibitor (Complete Mini; Roche). After centrifugation, the nuclear protein was extracted from the treated supernatants.

Western blot analysis was carried out as described previously (Sekikawa *et al*, 2005). Briefly, protein extract (12.5 *μ*g) was fractionated by sodium dodecyl sulphate-polyacrylamide gel electrophoresis and transferred to a polyvinylidene difluoride membrane. The membrane was incubated with primary antibodies and then with a peroxidase-conjugated secondary antibody. Proteins were detected by an enhanced chemiluminescence system (Amersham Pharmacia Biotech, Buckinghamshire, UK).

### Statistical analysis

The *χ*^2^ test was performed to determine correlations among the various parameters, and Fisher's exact test was also used, as necessary. Cumulative survival rate was assessed by the Kaplan–Meier method and analysed by log-rank test. Multivariate analysis was performed with the Cox proportional hazards model with hazard ratios (HRs) and 95% confidence intervals (CIs) to evaluate independent prognostic factors. Differences at *P*<0.05 were considered statistically significant.

## RESULTS

### Expression of SDF-1*α* in normal colon and CRC tissues

**Figure 1:**
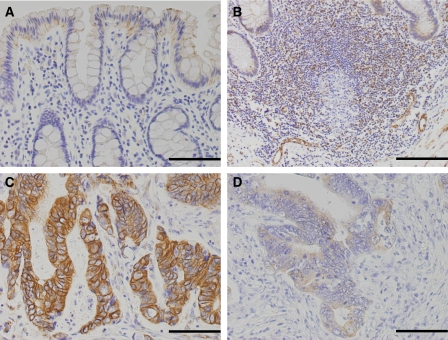
Immunohistochemistry for SDF-1*α* in the normal colon and in CRC tissues. (**A**) Weak to negative immunoreactivity is observed in the cytoplasm of non-neoplastic epithelial cells. (**B**) Immunoreactivity for SDF-1*α* is also observed in the lymphoid follicle in the colonic mucosa. (**C**) Strong type: immunoreactivity is strongly detected in the cytoplasm of cancer cells. (**D**) Weak type: immunoreactivity is weakly detected in the cytoplasm of cancer cells. Immunoreactivity is also observed in the endothelial cells of the tumour stroma. Scale bars=100 *μ*m.

In normal colorectal epithelium adjacent to the tumour, weak to negative immunoreactivity of SDF-1*α* was observed in the cytoplasm of non-neoplastic cells (Figure 1A). Stromal cell-derived factor-1*α* immunoreactivity was also observed in the lymphoid follicles in the colonic mucosa (Figure 1B).

In the CRC tissues, SDF-1*α* immunoreactivity was detected in the cytoplasm of cancer cells and vascular endothelial cells in the tumour stroma (Figure 1C and D). Thirty-eight (63.3%) of the 60 CRCs showed strong immunoreactivity for SDF-1*α* at the invasive front and were classified as strong type. Seventeen CRCs showed weak to faint SDF-1*α* immunoreactivity. Five showed no signal and were subsequently also classified as weak type.

### Relationship between SDF-1*α* expression and clinicopathological features in CRCs

Tumour stage and prevalence of lymphatic invasion, venous invasion, and LN metastasis were significantly higher in CRCs showing strong SDF-1*α* expression than in those with weak expression (Table 2). However, none of the other parameters – age, gender, tumour location, tumour size, or differentiation – had a significant relationship to SDF-1*α* expression.

### Expression of CXCR4 in normal colon and CRC tissues

**Figure 2:**
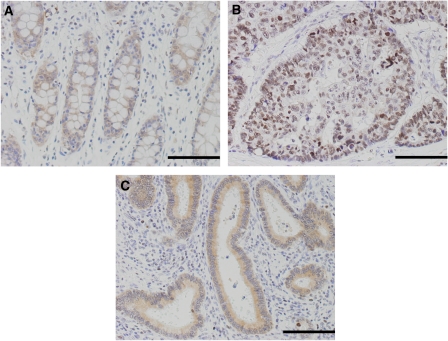
Immunohistochemistry for CXCR4 in the normal colon and in CRC tissues. (**A**) Weak immunoreactivity is observed in the cytoplasm and plasma membrane of non-neoplastic epithelium cells. (**B**) Nuclear type: immunoreactivity is detected in the cytoplasm of cancer cells weakly and in the nuclei strongly. (**C**) Cytomembrane type: immunoreactivity is detected in the cytoplasm and plasma membrane of cancer cells. Scale bars=100 *μ*m.

In the normal colorectal epithelium adjacent to the tumour, weak immunoreactivity for CXCR4 was generally detected in the cytoplasm and plasma membrane of non-neoplastic epithelial cells (Figure 2A).

In the CRC tissues, CXCR4 immunoreactivity was found not only in cancer cells but also in lymphocytes in the tumour stroma. Here, we focussed on the immunostaining pattern for CXCR4 in cancer cells at the invasive fronts of tumours. Forty-seven (78.3%) of the 60 CRCs were positive for CXCR4 expression at the invasive front. In 29 (61.7%) of these 47 CXCR4-positive CRCs, CXCR4 immunoreactivity was clearly localised in the nucleus (nuclear type; Figure 2B). In the remaining 18 cases (38.3%), it was detected diffusely in the cytoplasm and plasma membrane (cytomembrane type; Figure 2C).

### Localisation of CXCR4 proteins in the CRC cell line

**Figure 3:**
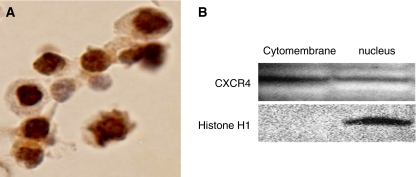
Subcellular localisation of CXCR4 in the CRC cell line HT29. (**A**) Immunohistochemistry. CXCR4 immunoreactivity is detected not only weakly at the plasma membrane but also strongly in the nuclei. (**B**) Western blot analysis. CXCR4 immunoreactivity is detected not only in the cytoplasmic and membranous (‘cytomembrane’) fraction but also in the nuclear (‘nuclear’) protein fraction from HT29 cells.

We examined the specificity of CXCR4 immunoreactivity by using the CRC cell line HT29. CXCR4 immunoreactivity was detected not only weakly at the plasma membrane but also strongly on the nucleus strongly (Figure 3A).

Localisation of CXCR4 proteins was also examined in HT29 cells by western blot analysis. Western blots revealed CXCR4 protein in not only the cytomembrane fraction but also the nucleus (Figure 3B).

### Relationship between CXCR4 expression and clinicopathological features in CRCs

CXCR4 expression was significantly positive in CRCs with high tumour stage and with LN metastasis (Table 3). Additionally, CXCR4 expression positivity tended to be higher in CRCs with lymphatic invasion than in those without.

We next divided CXCR4-positive CRCs into nuclear and cytomembrane types and further investigated the importance of CXCR4 immunostaining patterns (Table 4). Comparison of nuclear- and cytomembrane-type CXCR4-positive CRCs revealed that the nuclear-type CRCs were significantly more likely to show poor differentiation, high tumour stage, and frequent LN metastasis. In addition, lymphatic invasion tended to be more frequent in nuclear-type CRCs. Moreover, comparison of CXCR4-negative and CXCR4 nuclear-type-positive CRCs revealed that malignant potential, as indicated by tumour stage, lymphatic invasion, venous invasion, and LN metastasis, was clearly higher in CXCR4 nuclear-type-positive tumours.

### Relationship between SDF-1*α* and CXCR4 expression in primary CRCs

CXCR4-positive CRCs showed significantly stronger SDF-1*α* expression than did negative ones (Table 5; *P*=0.002). However, among the CXCR4-positive CRCs, SDF-1*α* expression did not differ between the nuclear and cytomembrane types.

### Expression of SDF-1*α* and CXCR4 in metastasised tumour cells in LNs

**Figure 4:**
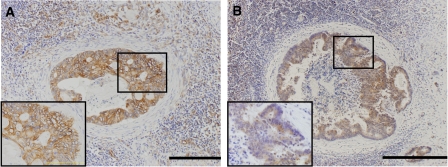
Expression of SDF-1*α* and CXCR4 in MTLNs. Representative photomicrographs of MTLNs, showing (**A**) strong SDF-1*α* expression and (**B**) cytomembrane-type CXCR4. Scale bars=100 *μ*m.

Twenty-seven CRCs showed metastasis to their related LN. Among these primary CRCs, 23 (85.2%) had strong expression of SDF-1*α*. Twenty-four (88.9%) of the 27 metastasised tumours in LNs (MTLNs) showed strong expression of SDF-1*α*. Thus, most of the CRCs with LN metastases (*n*=22, 81.5%) showed strong SDF-1*α* expression, not only in the primary lesion but also in their MTLNs (Figure 4A).

**Figure 5:**
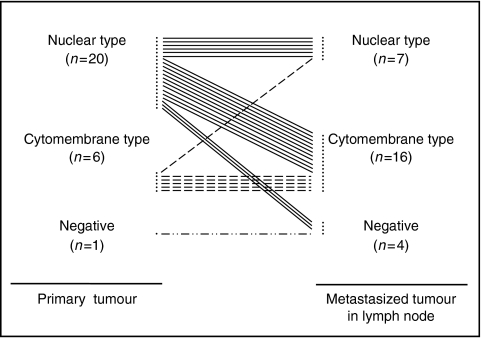
Immunostaining pattern of CXCR4 in primary CRCs and MTLNs. MTLNs show significant cytomembranous immunoreactivity even when the primary tumours show nuclear immunoreactivity (*n*=27, *P*=0.012).

Twenty-six (96.3%) of the 27 primary CRCs with LN metastases were positive for CXCR4 expression: 20 (74.1%) with nuclear patterns and 6 (22.2%) with cytomembrane patterns (Figure 5). Twenty-three (85.2%) of the 27 MTLNs were positive for CXCR4 expression. Sixteen (69.6%) of the MTLNs had CXCR4 immunostaining patterns of the cytomembrane type and seven (30.4%) were of the nuclear type. Interestingly, 11 (55.0%) of the 20 primary CRCs that had nuclear-type CXCR4 immunoreactivity had cytomembrane-type CXCR4 positivity in their LNs. However, five (83.3%) of the six CRCs that had cytomembrane-type CXCR4 immunoreactivity had the same CXCR4 immunoreactivity patterns in their MTLNs. These findings suggest that MTLNs are significantly more likely to show cytomembrane pattern CXCR4 immunoreactivity even when their primary CRCs show nuclear-type CXCR4 immunoreactivity (Figure 4B).

### Survival analysis

Log-rank statistics showed that lymphatic invasion, LN metastasis, and disease stage were significant prognostic indicators for overall patient survival (*P*=0.043, 0.0005, and <0.0005, respectively). There was no significant correlation between prognosis and other clinicopathological features.

**Figure 6:**
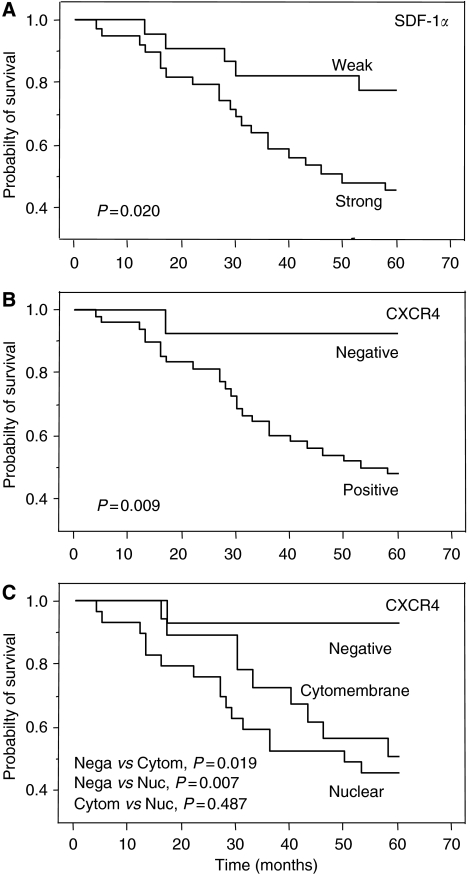
Overall survival according to (**A**) SDF-1*α* immunoreactivity; (**B**) CXCR4 immunoreactivity; and (**C**) CXCR4 immunostaining pattern in patients with CRCs (*n*=60). Kaplan–Meier survival curves were constructed and pairwise differences were analysed by log-rank test. Nega, negative; Cytom, cytomembrane; Nuc, nuclear.

To assess the prognostic significance of SDF-1*α* and CXCR4 expression, Kaplan–Meier survival curves were constructed. The prognosis of CRC patients with strong SDF-1*α* expression was significantly worse than that of CRC patients with weak SDF-1*α* expression (Figure 6A). Patients with CXCR4-positive CRCs also had significantly worse outcomes than those with CXCR4-negative ones (Figure 6B). Furthermore, CRC patients with nuclear-type CXCR4 expression tended to have worse outcomes than those with cytomembrane-type expression (Figure 6C).

Multivariate analysis by Cox regression and correction for disease stage, CXCR4 expression, and SDF-1*α* expression showed that stage was an independent prognostic factor (HR=2.63, 95% CI 1.08–6.41, *P*=0.033), whereas SDF-1*α* (HR=1.46, 95% CI 0.52–4.10, *P*=0.475) and CXCR4 expression (HR=5.08, 95% CI 0.65–40.0, *P*=0.123) were not statistically significant.

## DISCUSSION

CXCR4 has been shown experimentally to be crucial for cancer cell adhesion and/or migration (Kawamata *et al*, 2003), suggesting the involvement of CXCR4 in tumour invasion and metastasis. Supporting such *in vitro* data, our clinicopathological study revealed that CXCR4 expression was significantly positive in CRCs with a high tumour stage and LN metastasis, and patients with CXCR4-positive CRCs showed a significantly worse outcome than those in whom CRCs were negative, suggesting that CXCR4 is a significant prognostic marker in CRC patients.

Interestingly, we found that CRC cells had two distinct immunostaining patterns for CXCR4: cytomembrane and nuclear types, similar to those reported in cases of hepatocellular carcinoma (Shibuta *et al*, 2002), breast cancer (Kato *et al*, 2003; Shim *et al*, 2006), lung cancer (Spano *et al*, 2004), and nasopharyngeal carcinoma (Wang *et al*, 2005). As CXCR4 is a transmembrane protein, it was originally believed that its expression would be detectable at the plasma membrane and in the cytoplasm, and in contrast, that nuclear CXCR4 expression would be detected nonspecifically by immunohistochemistry. However, our initial examination of the localisation of CXCR4 in CRC cell lines showed that CXCR4 immunoreactivity was detectable not only in the cytomembrane but also in nuclear protein fractions, suggesting the specificity of CXCR4 immunoreactivity in the nuclei of CRC cells. Of note, CRC patients with nuclear-type CXCR4 expression showed more frequent LN metastasis, poor differentiation, and worse outcome than those with cytomembrane-type expression, suggesting that nuclear expression of CXCR4 may play a role in the progression of CRC.

Although a few *in vitro* studies have described SDF-1*α* expression in CRC cell lines (Brand *et al*, 2005; Wendt *et al*, 2006), ours is the first demonstration that considerable numbers of human CRC lesions indeed produce SDF-1*α*. Moreover, it was important that SDF-1*α* expression was associated with not only lymphatic invasion, venous invasion, and LN metastasis but also survival of CRC patients. In conflict with our present data, a single study by northern blot preliminarily reported that *SDF-1α* mRNA expression was decreased in CRC tissues in comparison with normal colon tissues (Shibuta *et al*, 1997). However, we found that SDF-1*α* was moderately produced in the lymphoid follicles but faintly by colorectal epithelial cells in normal colon tissues. Thus, northern blot analysis of CRC tissues might not compare the *SDF-1α* mRNA levels of normal colorectal epithelial cells and CRC cells. Although CRC tissues also contain SDF-1*α*-positive stromal cells, immunohistochemistry clearly demonstrated that the cancerous cells in more than 50% of CRC samples examined had much stronger expression of SDF-1*α* than their neighbouring normal colonic epithelial cells. Accordingly, as with CXCR4 overexpression, the SDF-1*α*–CXCR4 axis appears to play important roles in the progression of CRC.

One concern is the molecular mechanism of nuclear CXCR4 expression and the pathobiological roles of the SDF-1*α*–CXCR4 axis in CRC progression. Stromal cell-derived factor-1*α* stimulation triggers CXCR4 internalisation, involving G-protein-coupled receptor kinases, followed by the binding of *β*-arrestin and subsequently CXCR4 endocytosis (Haribabu *et al*, 1997; Orsini *et al*, 1999; Cheng *et al*, 2000; Burger *et al*, 2003). On the other hand, Zeelenberg *et al* (2003) showed that endogenous SDF-1*α* binds newly synthesised CXCR4 and subsequently inhibits the translocation of CXCR4 to the cell surface. In these contexts, we expected that SDF-1*α* expression might be associated with nuclear CXCR4 expression in CRC cells, and therefore investigated the CXCR4 expression pattern in CRC cells that had metastasised to LNs, as SDF-1*α* is abundantly produced and furthermore, most MTLNs showed strong SDF-1*α* expression there. However, contrary to expectation, even CRC patients with nuclear patterns of CXCR4 expression in the primary lesion frequently had cytomembrane-type CXCR4-positive tumours in the LNs. Moreover, we observed no significant relationships between SDF-1*α* expressional intensity and CXCR4 expression pattern in the primary CRCs. Thus, considering these findings as a whole, it is difficult to explain the molecular mechanism of nuclear CXCR4 expression in CRC cells in terms of its association with SDF-1*α* alone. On the other hand, it is known that hypoxia-inducible factor-1*α* (HIF-1*α*) plays a critical role in CXCR4 expression in the tumour cells under hypoxic condition (Pouysségur *et al*, 2006). Interestingly, Shim *et al* (2006) have recently reported that HIF-1*α* expression is reduced in MTLNs of patients with breast cancer and moreover that MTLNs showed cytoplasmic immunoreactivity for CXCR4 expression. Although it is still unclear why immunostaining pattern of CXCR4 in MTLNs is different from that in primary lesions, SDF-1*α* and HIF-1*α* may be possible keys to determine CXCR4 expression pattern.

In summary, we demonstrated here that both CXCR4 and SDF-1*α* expressions are closely associated with LN metastasis and poor prognosis in patients with CRC. Moreover, we clarified the fact that CXCR4 is detectable not only at the plasma membrane but also in the cytoplasm or nucleus of CRC cells, although the molecular mechanism of nuclear CXCR4 expression remains to be elucidated. As nuclear CXCR4 expression in the primary CRC may reflect increased potential for LN metastasis, the pathobiological significance of translocated CXCR4 needs to be investigated in future studies.

## Figures and Tables

**Table 1:** Clinicopathological features of the patients with colorectal cancer

*Gender*	
Man	41 (68.3%)
Woman	19 (31.7%)
	
Age (years, mean±s.d.)	63.8±10.9 (39–88)
	
*Tumour location*	
Colon	49 (81.7%)
Rectum	11 (18.3%)
	
Tumour size (cm, mean±s.d.)	4.1±2.0 (0.7–8.7)
	
*Differentiation*	
Well	26 (43.3%)
Mod	30 (50.0%)
Por	4 (6.7%)
	
*UICC stage*	
I	10 (16.7%)
II	23 (38.3%)
III	12 (20.0%)
IV	15 (25.0%)
	
*Lymphatic invasion*	
None	15 (25.0%)
Present	45 (75.0%)
	
*Venous invasion*	
None	28 (46.7%)
Present	32 (53.3%)
	
*Lymph node metastasis*	
None	33 (55.0%)
Present	27 (45.0%)

**Table 2:** Relationship between SDF-1*α* expression and clinicopathological features

	**SDF-1*α* expression**		
	**Weak (*n*=22)**	**Strong (*n*=38)**	***P*-value**
*Gender*			
Man	12	29	0.145
Woman	10	9	
			
Age (years, mean±s.d.)	63.7±10.7	63.9±11.2	0.962
			
*Tumour location*			
Colon	18	31	0.999
Rectum	4	7	
			
Tumour size (cm, mean±s.d.)	3.6±2.4	4.4±1.8	0.128
			
*Differentiation*			
Well	12	14	
Mod	9	21	0.185
Por	1	3	
			
*UICC stage*			
I	8	2	
II	10	13	0.0002
III	3	9	
IV	1	14	
			
*Lymphatic invasion*			
None	11	4	0.001
Present	11	34	
			
*Venous invasion*			
None	15	13	0.016
Present	7	25	
			
*Lymph node metastasis*			
None	18	15	0.003
Present	4	23	

**Table 3:** Comparison of the clinicopathologial features between the CXCR4-positive and CXCR4-negative CRC patients

	**Positive (*n*=47)**	**Negative (*n*=13)**	***P*-value**
*Gender*			
Man	31	10	0.522
Woman	16	3	
			
Age (years, mean±s.d.)	64.0±10.8	63.2±11.9	0.829
			
*Tumour location*			
Colon	38	11	0.999
Rectum	9	2	
			
Tumour size (cm, mean±s.d.)	4.3±1.8	3.4±2.8	0.158
			
*Differentiation*			
Well	20	6	
Mod	24	6	0.872
Por	3	1	
			
*UICC stage*			
I	3	7	
II	18	4	<0.0001
III	11	2	
IV	15	0	
			
*Lymphatic invasion*			
None	9	6	0.070
Present	38	7	
			
*Venous invasion*			
None	19	9	0.115
Present	28	4	
			
*Lymph node metastasis*			
None	21	12	0.004
Present	26	1	
			

**Table 4:** Relationship between CXCR4 immunostaining pattern and clinicopathological features in patients with CXCR4-positive CRC

	**Nuclear (*n*=29)**	**Cytomembrane (*n*=18)**	***P*-value**
*Gender*			
Man	19	12	0.999
Woman	10	6	
			
Age (years, mean±s.d.)	63.0±11.4	65.6±9.9	0.419
			
*Tumour location*			
Colon	21	17	0.999
Rectum	8	1	
			
Tumour size (cm, mean±s.d.)	4.3±1.7	4.2±2.0	0.882
			
*Differentiation*			
Well	9	11	
Mod	17	7	0.028
Por	3	0	
			
*UICC stage*			
I	0	3	
II	9	9	0.022
III	9	2	
IV	11	4	
			
*Lymphatic invasion*			
None	3	6	0.068
Present	26	12	
			
*Venous invasion*			
None	9	10	0.172
Present	20	8	
			
*Lymph node metastasis*			
None	9	12	0.036
Present	20	6	
			

**Table 5:** Relationship between CXCR4 expression and SDF-1*α* expression in primary tumour

	**CXCR4 positive**			
	**Nuclear**	**Cytomembrane**	**Total**	**CXCR4 negative**
*SDF-1α expression*				
Weak	7	5	12	10
Strong	22	13	35^a^	3

a CXCR4-positive CRCs showed significantly stronger SDF-1*α* expression than did negative ones (*P*=0.002).
